# Meniscal rim instability has a high prevalence and a variable location

**DOI:** 10.1007/s00402-023-04908-9

**Published:** 2023-05-19

**Authors:** Nora Ammann, Raphael Kaelin, Elias Ammann, Erich Rutz, Kathrin Studer, Victor Valdarrabano, Carlo Camathias

**Affiliations:** 1 Medical School Basel, University of Basel, Basel, Switzerland; 2 Department of Surgery, Kantonsspital Graubünden, Loëstrasse 170, 7000 Chur, Switzerland; 3 Practice LEONARDO, Hirslanden Klinik Birshof, Münchenstein, Switzerland; 4 Department of Orthopedic Surgery, Spital Thurgau AG, Frauenfeld, Switzerland; 5 Department of Orthopedics, The Royal Children’s Hospital, The University of Melbourne, Melbourne, Australia; 6 Praxis Zeppelin, St. Gallen, Switzerland; 7 Orthopedic Departement, Swiss Ortho Center, Schmerzklinik Basel, Basel, Switzerland

**Keywords:** Meniscal rim instability, Meniscal rim stability, Lateral discoid meniscus

## Abstract

**Introduction:**

Most classification systems for lateral discoid meniscus do not evaluate instability of the meniscal peripheral rim. Considerable variability in the prevalence of peripheral rim instability has been published, and it appears that instability is underestimated. The purpose of this study was: first, to evaluate the prevalence of peripheral rim instability and its location in the symptomatic lateral discoid meniscus, and second, to investigate if patient age or type of discoid meniscus are possible risk factors for instability.

**Methods:**

A cohort of 78 knees that underwent operative treatment due to symptomatic discoid lateral meniscus was analyzed retrospectively for the rate and location of peripheral rim instability.

**Results:**

Out of the 78 knees, 57.7% (45) had a complete and 42.3% (33) had an incomplete lateral meniscus. The prevalence of peripheral rim instability in symptomatic lateral discoid menisci was 51.3%, and with 32.5%, the anterior attachment was most commonly affected, followed by the posterior (30%) and central (10%) attachment. 27.5% of the tested menisci were unstable anteriorly and posteriorly. There was no significant difference in the prevalence of rim instability between the type of discoid menisci (complete vs. incomplete), nor was there a significant correlation for age as a risk factor for instability.

**Conclusion:**

The discoid lateral meniscus has a high prevalence and variable location of peripheral rim instability. Meniscal rim stability must be tested and addressed cautiously in all parts and in all types of discoid lateral menisci during operative treatment.

## Introduction

Peripheral rim instability of discoid menisci is not a primary issue in the most popular Watanabe classification system. This classification instead focuses on the size of the meniscus than on stability. The only unstable type (Wrisberg type meniscus) is rarely found. However, Klingele et al. described in their cohort of symptomatic knees with a discoid lateral meniscus a prevalence of 28% in peripheral rim instability and the complete type and young age as risk factors for instability. Furthermore, they reported instability in the posterior root, the anterior horn, and the central part of the discoid meniscus [1]. Moreover, Good et al. focused on the outcome after arthroscopic treatment. However, they assessed an instability rate of 77%, most frequently in the anterior part. Consequently, they proposed a new classification system covering meniscal size, instability, and location [2]. According to these reports, instability appears more frequently. However, therapy still consists of a consensus to restore the normal anatomy of the lateral meniscus by performing an arthroscopic central saucerization. Therefore, instability might be underestimated. Since an unstable lateral meniscus increases tibiofemoral contact pressure and contributes to rotational instability [3–5], it seems crucial to meticulously probe the meniscus’ rim stability and repair if unstable, even if there is no visible tear.

Since we conscientiously tested instability in these patients, we questioned how frequent rim instability is in our patient cohort. Therefore, this study aimed to elucidate the prevalence of rim instability in symptomatic discoid lateral menisci in children and adolescents. We investigated the patient cohort treated at our institution for rim instability, location of instability, and predisposing factors. We hypothesized that peripheral rim instability is present in more than 30% of children with a symptomatic lateral discoid meniscus and is an underestimated problem. Further, a complete discus was presumed to be a predisposing factor for rim instability.

## Material and methods

The local ethical committee approved this retrospective study. All patients who underwent surgical treatment for a symptomatic discoid lateral meniscus between 01/2008 and 04/2013 were identified by screening the operation plan. All patients had lateral compartment pain or mechanical symptoms and MRI confirming a discoid lateral meniscus prior to surgery. All patients were treated by the same surgeon.

Exclusion criteria were: incomplete files, previous trauma to the knee, asymptomatic, but incidentally found discoid meniscus.

All patient’s operative reports, intraoperative photos, and videos were analyzed. Meniscal characteristics were assessed regarding the type (complete vs. incomplete), stability, and location of possible rim instabilities. Meniscal stability was assessed in all peripheral parts of the meniscus with a meniscal probe after central saucerization. We defined a meniscus as unstable, according to Camathias et al., when it was probed under direct vision in a 90° flexed knee and compared with the rest of the meniscus and the ipsilateral medial or lateral meniscus. Increased mobility of more than 5 mm was considered unstable [6], and the location of the instability was noted.

It was analyzed if patient age or type of discoid meniscus (complete vs. incomplete) were risk factors for peripheral rim instability.

Statistical analysis was performed with a standard statistical software package (JMP, version 12; SAS Institute, Cary, NC). The *χ*^2^ test was used for nominal data, and all continuous data were normally distributed and compared using a *t* test or ANOVA, and corrected with Tuckey-Kramer HSD.

The level of statistical significance was set at *P* < 0.05.

## Results

A total of 78 knees of 62 patients (26 female) were included (16 bilateral cases) with a mean age at the treatment of 9.9 years ± 3.1 SD (standard deviation) ranging from 3 to 16 years. Surgery was performed in 36 left and 42 right knees, with 45 complete and 33 incomplete discoid lateral menisci (57.7% complete vs. 42.3% incomplete).

In 40 out of 78 knees, rim instability was detected (51.3%). Most frequently, the anterior attachment (32.5%, 13 out of 40) was unstable, followed by the posterior and central attachment with 30% (12 out of 40) and 10% (4 out of 40), respectively. 11 discoid menisci (27.5%) were unstable at both, the anterior and posterior attachment.

Incomplete discoid menisci were more frequently unstable with 63.6% (21 out of 33) compared to the complete type with a rate of 42.2% (19 out of 45); however, the difference was statistically not significant (*p* = 0.06, *χ*^2^ test). Further, no difference in instability location was found between incomplete and complete discoid meniscus (*p* = 0.19, *χ*^2^ test). As well, incomplete and complete discoid meniscus did not differ significantly regarding age (*p* = 0.94, *t*-test) and there was no significant difference in age between localizations of instability (*p* = 0.34, ANOVA).

The mean age of patients with rim instability was 9.8 years compared to 10.1 years for those with stable menisci (*p* = 0.7, *t*-test).

## Discussion

The most important finding of this study was that more than half of all symptomatic discoid lateral meniscuses were unstable. With the present controversial data, this still may be underestimated. Most of them were unstable anteriorly or posteriorly, followed by multifocal instability. Further, the rate of instability did not differ significantly with meniscus size (incomplete vs. complete).

The rate of instability in the present study is higher than in most former studies. Moreover, a higher rate of multifocal instability was detected. Klingele et al. published an instability rate of 28%, located posteriorly in 38.9%, central in 11.1%, and 47.2% of cases at the anterior horn [1]. The high proportion of anterior meniscus instability stands following the present study, whereas multifocal instability was even more common. Good et al. reported in a small cohort peripheral discoid meniscus detachment in 77% of cases, with the predominant location of instability in the anterior part [2]. Others documented peripheral meniscal instability after central saucerization in 33% and 42% of cases, respectively [7, 8], whereas the instability’s focus was mainly posterior.

In this study’s population, peripheral rim instability was not significantly associated with complete or incomplete discoid type. Notably, 64% of incomplete and 42% of complete discoid menisci were unstable, in contrast to former findings describing a higher rate of instability in complete types [1]. However, peripheral rim instability is not a problem, only occurring in complete-type discoid menisci. Therefore, every type of symptomatic discoid meniscus needs meticulous probing of stability in all its parts during surgery.

Conversely to the medial meniscus, which has been widely investigated regarding stress distribution, less is known about the biomechanical properties of the lateral meniscus. However, several biomechanical studies reported increased tibiofemoral contact pressure due to posterior meniscal instability [3, 9]. Even anterior instability of the lateral meniscus can raise lateral compartment peak forces [10]. Moreover, the lateral meniscus is a secondary restraint in anterior knee instability. However, solitary lateral meniscus instability leads to increased rotation movement in the knee joint [5]. With a non-functional secondary knee stabilizer and more rotation, the strain on the anterior crucial ligament as the primary stabilizer increases. As such, an insufficiently treated discoid meniscus with remaining instability might predispose to a ligamentous injury in a traumatic event. This notion is fundamental considering the young age and activity of patients. Further, the saucerized meniscus must be protected from tearing due to an unphysiological high range of knee rotation.

The high proportions of discoid meniscus rim instability and the variability of the instability’s location emphasize the importance of stability testing during arthroscopic treatment. Moreover, these findings point out that the Watanabe classification does not comprehensively describe the discoid meniscus pathology for clinical decision-making as rim instability is not only a problem of the posterior attachment and anterior horn instability can be missed easily.

Regarding treatment, most surgeons approve central saucerization and stabilization where needed, concerning young patient age and long-term outcome. After total meniscectomy, degeneration of the joint is frequent. However, mild to moderate degenerative changes are frequently found after partial resection of a discoid lateral meniscus [11–13]. Although the clinical outcome is acceptable in most studies, it needs to be considered that follow-up periods of ten years might not be representative of long-term outcomes in children or adolescents. Therefore, restoring physiologic anatomy appears of the utmost importance to prevent osteoarthritis. Since peripheral rim instability might not be as apparent as a meniscal tear, stability testing of the meniscal rim and the meniscal-capsular junction and suture stabilization of all instabilities play an essential role in a conscientious treatment. Solitary saucerization and saucerization with an additional suture repair showed no differences in short- and mid-term clinical outcomes [14]. A small review agrees with those findings. However, the most extended follow-up was limited to 5.4 years [15]. In a three-year follow-up of a minor cohort, patients treated with saucerization and additional suture repair, Wasser et al. showed good clinical outcomes, low complication rate, and correlation with satisfying postoperative MRI findings [16].

Considering this, it seems that suture stabilization does not negatively affect the clinical outcome and might help prevent the development of osteoarthritis.

Although meniscal rim stabilization may not be beneficial in the short- and mid-term, the effect of an unstable lateral meniscus might be detrimental in the longer term.

To our knowledge there are still no published studies with long-term data after surgical treatment of discoid lateral meniscus and so the benefit of suturing in case of peripheral rim instability is not proven yet. Concerning the patients’ young age, long-term results are crucial and further studies are required.

This study has several limitations, most of which are related to the retrospective design. However, the same surgeon treated all patients at the same institution, so uniform documentation was available for all patients. All operative reports contained detailed documentation of the pathology and stability of the meniscus, and intraoperative images and video were evaluated for each patient. This study included only symptomatic children with a mean age of 9.9 years, similar to former publications focusing on children with symptomatic discoid meniscus [1, 2, 8]. The findings may not apply to adult patients with a discoid meniscus who might be symptomatic due to a tear rather than meniscal instability without a tear.

Further, patients’ outcome was not included and was not the focus of this study. However, clinical outcome in the mid-term has been published widely and is good in most patients.

## Conclusions

The discoid lateral meniscus has a high prevalence and variable location of peripheral rim instability. Meniscal rim stability must be tested and addressed cautiously in all parts and in all types of discoid lateral menisci during operative treatment.

## Data Availability

The datasets generated and analyzed during this study are available from the corresponding author on reasonable request.
